# The phylogenetic analysis of the new emerging ALV-K revealing the co-prevailing of multiple clades in chickens and a proposal for the classification of ALV-K

**DOI:** 10.3389/fvets.2023.1228109

**Published:** 2023-07-28

**Authors:** Jinhan Guo, Qiaomu Deng, Weiyu Zhu, Fumei Fu, Linmin Liu, Tianchao Wei, Ping Wei

**Affiliations:** ^1^Institute for Poultry Science and Health, Guangxi University, Nanning, China; ^2^College of Animal Science, Guizhou University, Guiyang, China

**Keywords:** subgroup K avian leukosis virus, molecular epidemiology, phylogenetic analysis, classification, genetic distance, clades

## Abstract

Subgroup K avian leukosis virus (ALV-K) is a new subgroup of avian leukosis virus (ALV) that was first defined in 2012 and has been become prevalent in Chinese native chickens in recent years. An in-depth analysis of the genetic diversity of ALV-K was performed in the study. By Blast analysis, the *env* gene and the sequences of the 25 ALV-K isolates we isolated were found to be closely related to the isolates from Guangdong, Hebei, Jiangsu, and Hubei provinces, China. Further eighty-nine sequences of the gp85 gene of ALV-K strains available were used in the phylogenetic and genetic distance analyses for the classification. ALV-K was divided into two second-order clades (Clades 1.1 and 1.2) and three third-order clades (Clades 1.2.1, 1.2.2, and 1.2.3), indicating that not only 1.1 and 1.2.3, the two old clades which are prevalent in Japan, but also two new clades (1.2.1, 1.2.2), are co-prevalent in China. The representative strains of each clade were defined for the first time. Notably, Clade 1.2.2 was found to have a deletion of an amino acid residue in the gp85 gene, which was obviously different from Clades 1.1, 1.2.1, and 1.2.3. The proposed classification method will facilitate future studies of ALV-K epidemiology and the comparison of sequences obtained across the world. The first global comprehensive molecular epidemiological analysis was accomplished on the emerging ALV-K.

## 1. Introduction

Avian leukosis (AL) is a disease caused by the avian leukosis virus (ALV), which causes huge economic losses to the poultry industry worldwide. ALV belongs to the genus Alpharetrovirus of the family Retroviridae (1). So far, ALV has been divided into 11 subgroups according to the differences in envelope protein (ENV) (2). Subgroup J ALV (ALV-J) is currently the most common and dominant exotic subgroup in China, while subgroups A and B are less common and subgroups C and D are rare (3, 4). It is worth noting that despite the fact that ALV-K has been prevalent in China for over a decade, there are still no systematic epidemiological studies on it.

Initially, in 2012, a novel subgroup of ALV named ALV-K was isolated from a local chicken breed (Luhua) in Jiangsu Province, China (5). In previous studies, an exogenous ALV strain TW-3593 from Chinese Taiwan in 2008 (6) and several fowl glioma viruses (FGV) isolated from Japan (7–10) were reported to be from indigenous chickens, which showed high amino acid (aa) sequence similarity in the gp85 gene (more than 90%) with the Chinese ALV-K isolates but showed low similarity with other ALVs (11). Subsequently, ALV-K isolates were discovered in Yellow-chicken in Guangdong and Shandong in China (12). Recently, our group found that ALV-K is widespread and prevalent in Yellow-chicken in Guangxi province in southern China.

In general, early isolates of ALV-K exhibited no or low pathogenicity. Chen et al. (13) reported that key aa sites 199–205, 269, 319, 321, and 324 of ALV-K env contributed to the weaker replication capacity of ALV-K than that of ALV-A. This is the first time that molecular factors for the weak replicative ability of ALV-K have been revealed. Zhao et al. (14) reported that strain GD14LZ (ALV-K) replicates more slowly in DF-1 cells than strains GD13, GD08 (ALV-A), and CHN06 (ALV-J). However, Liang et al. (15) reported that the ALV-K strain HB2015032 isolated from Layer in 2015 has exhibited tumorigenicity. These newly discovered ALV-K strains could be adapting in the Chinese local chickens, increasing their prevalence and pathogenicity in flocks. In addition, for routine eradication and diagnostic assays, the presence of ALV-K is not easily detected due to its slow replication compared to other subgroups and the fact that it is only cultured on cells for one passage, thus making it easy to miss detection (12–14). This virus may have existed in Chinese local chickens for a long time and probably as a persistent infection without notice, due to its weak replication ability (12, 14).

This will make the prevention, control, and eradication of ALV-K increasingly difficult and become a new potential threat to the Chinese poultry industry. In addition, for genetic evolution studies, the gp85 gene is commonly used for molecular epidemiological analysis, and it is also the most detailed sequence available for ALV-K in GenBank.

In the study, a total of 25 (*n* = 25) ALV-K isolates were newly identified recently in 2022–2023. Molecular characteristics and phylogenetic analysis of the ALV-K strains including these new isolates and all the strains available in the GenBank were performed, and a total of 89 sequences of the gp85 gene with known sampling information (i.e., time, geographic source, and host) were retrieved. The aim of our study was to gain systematic insights into the current epidemiology of ALV-K strains.

## 2. Materials and methods

### 2.1. Samples

A total of 14 clinical tissue samples and 1,500 plasma samples from nine commercial Yellow-chicken farms in Nanning, Guilin, Yulin, Guigang, Beihai, and Hechi of Guangxi Province, China, were collected during the years 2022–2023. Among them, anticoagulation blood samples were collected aseptically, and then were further separated by centrifugation at 3000 r/min for 5 min to obtain the plasma homogenates, which were stored at −80°C. The AL suspected tumors (vascular organs with enlargement or/and tumor-like nodular etc. changes) tissue samples from the clinical diseased chickens were collected by the farms and sent to the laboratory, and were ground at a ratio of 1:3 with sterile PBS, and stored at −80°C.

### 2.2. Virus isolation, identification, and sequencing of *env* gene

A continuous line of chicken embryo fibroblast cells (DF-1) without endogenous ALV was used to isolate ALV. DF-1 cells were grown in Dulbecco’s modified Eagle’s medium (DMEM) (Invitrogen, Shanghai, China) supplemented with 8% fetal bovine serum (FBS) from Uruguay^1^ at 37°C under 5% CO_2_.The virus isolation was performed according to the method of Wang et al. (16).

For identification, ALV P27 antigen was detected with the ALV antigen kit (BioChek, Netherlands), and the proviral genomic DNA samples were extracted with the Tissue Genomic DNA Purification Kit (TianGen, Beijing, China) according to the manufacturer’s instructions from the ALV P27 antigen-positive cell cultures. The subgroup-specific PCR was used to identify each subgroup of ALV based on the method reported by Li et al. (17) (Supplementary Table S1). Further amplification of the *env* genes were performed on the ALV-K positive samples. Two pairs of sequencing primers were used to amplify the *env* gene according to the sequence published in GenBank and referenced in the literature (18) (Table 1). PCR was conducted in a 25 μL volume according to the manufacturer’s instructions for PrimeSTAR Max DNA Polymerase (Takara, Dalian, China). The conditions for PCR were as follows: 95°C 3 min; 95°C 15 s, 60°C 15 s, 72°C, 30 s/kb (32 cycles); and 72°C 5 min. The PCR product was displayed by electrophoresis using 1.2% agarose in a Tris-EDTA (TBE) buffer gel, purified using the Universal DNA Purification Kit (TianGen, Beijing, China), and then cloned into the pMD18-T vector (Takara, Dalian, China) for sequencing, as the previous description (16). The recombinant plasmids were transformed into competent cells of the *Escherichia coli* DH5α strain (TransGen, Beijing, China), and the positive clones were sent to sequence by the Shenzhen Huada Genomics Technology Service Co., Ltd. The sequences of the obtained *env* gene isolates were collated using DNA Star Lasergene 7.1 and then analyzed by BLAST.^2^

**Table 1:** Information of env gene primer sequences.

Primer	Sequence (5′-3′)	T_m_	Product size (bp)
ALV-KF1	CACCGATACAAAAACACTGGAGACC	60°C	1007/1010
ALV-KR1	CGGTAGCGAGGACCTGTCTGTG		
ALV-KF2	TCCAGGCCGCAACTCAC	60°C	1,214
ALV-KR2	CATACCACCACCCACGTACT		

### 2.3. Phylogenetic tree construction

Nucleotide sequences of the gp85 gene of 89 ALV-K strains, including 25 isolated from the study (GenBank accession numbers: OQ990388-OQ990412) and 64 from the National Center for Biotechnology,^3^ were used in the analysis (Supplementary Table S2). The reference strains of the ALV-K were isolated from Shandong (3), Guangdong (5), Hubei (1), Jiangsu (34), Hebei (5), Taiwan (1) in China, and Japan (15), respectively. Data processing and phylogenetic analysis were carried out with reference to the classification and nomenclature of Deng et al. (19, 20). Isolates with 100% sequence similarity were identified and removed. MAFFT v7.511 was used to align the dataset of ALV-K gp85 gene sequences. and the gp85 gene sequences were compared with the reference strain TW-3593 (6) and adjusted manually in BioEdit v7.2.5 (21, 22). The final alignment length was 1,008 nt. Four phylogenetic trees from four datasets (*n* = 38, 89, 69, and 12) using the gp85 gene were, respectively, constructed by using IQ-Tree v1.6.12 (23), following the steps of Deng’s method (19). The estimates of the average evolutionary distances were inferred using MEGA 11 (24).

### 2.4. Analyses of the amino acid (aa) sequences

Using the MUSCLE (Codons) multiple alignment method in MEGA 11, important aa residues encoded by the gp85 gene were compared between these isolates and the reference strains.

## 3. Results

### 3.1. Results of the BLAST analysis and the phylogenetic analysis

A total of 25 ALV-Ks were isolated during the years 2022–2023. The results of the analysis showed that the overall similarity of the *env* gene of these 25 viruses was 94–100%. Further the BLAST analysis, compared with the reference sequences in GenBank, showed that the Guangxi isolates were closely related to isolates from Guangdong, Hebei, Jiangsu, and Hubei (Supplementary Table S3).

**Figure 1:**
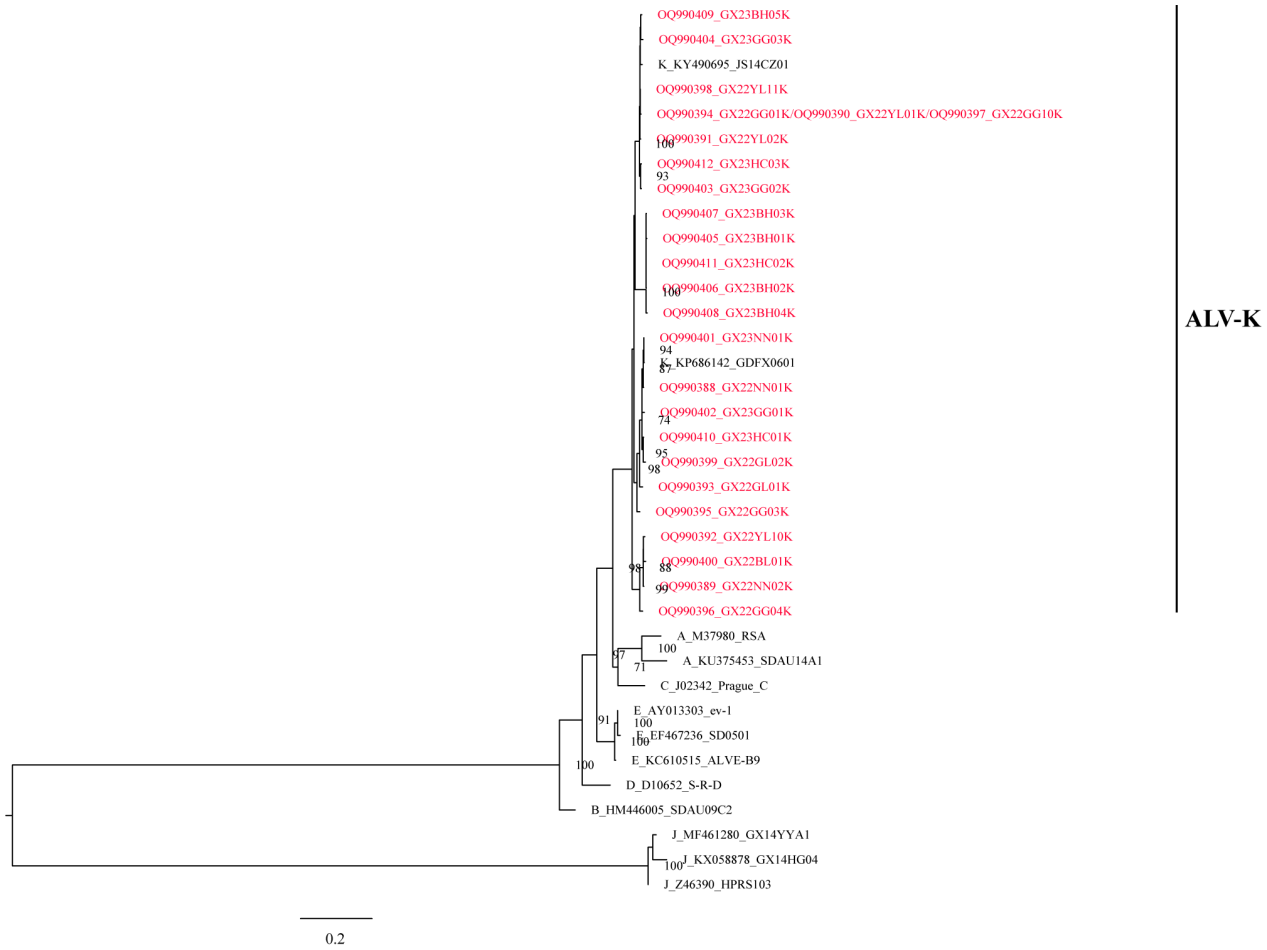
The phylogenetic analysis of ALV subgroups based on the gp85 gene.

All the above 25 isolates clustered in one evolutionary branch with ALV-K reference strains GDFX0601 and JS14CZ01, according to the analysis of ALV subgroups based on gp85 gene sequences, but not in any cluster with the reference strains from subgroups A-E, and were the most distantly related to the J subgroup reference strains, indicating that these 25 isolates belonged to ALV-K (Figure 1).

**Figure 2:**
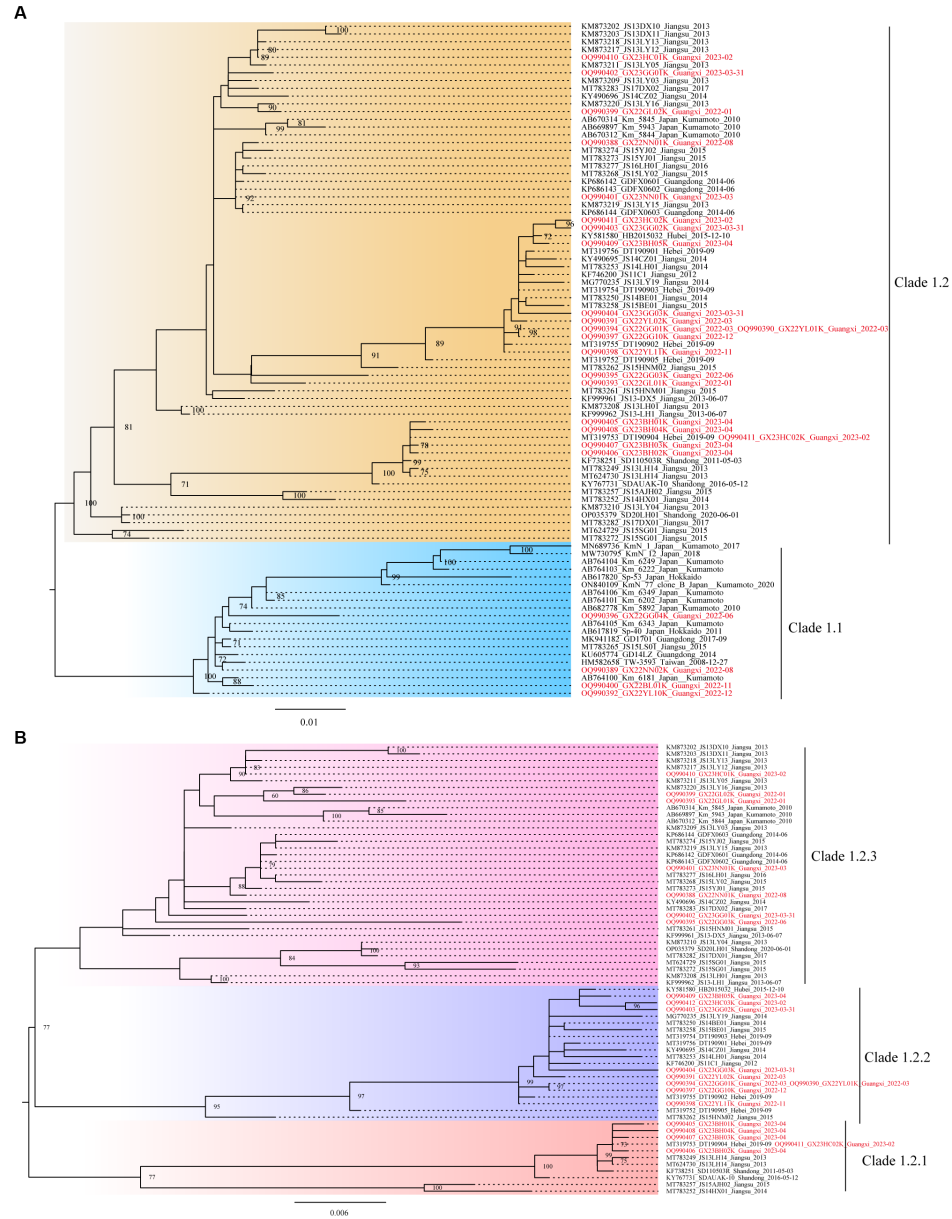
The phylogenetic trees constructed using the gp85 gene. **(A)** The analysis involved 87 nucleotide sequences from ALV-K. **(B)** The analysis involved 67 nucleotide sequences from dominant Clade 1.2 ALV-K.

Further, based on the available gp85 gene sequences, the phylogenetic analysis and genetic distance assessment of all the ALV-K strains revealed that ALV-K isolates were separated into two second-order clades (Clades 1.1 and 1.2; Figure 2A; Supplementary Table S4). Subdivision of one of the dominant branches, Clade 1.2, showed that these ALV-K isolates were divided into three third-order clades (Clades 1.2.1, 1.2.2, and 1.2.3; Figure 2B; Supplementary Table S4). The result showed that the Japanese isolates were distributed in Clades 1.1 and 1.2.3, while the Chinese isolates were distributed in Clades 1.1 and 1.2.1, 1.2.2, and 1.2.3 (Supplementary Figure S1).

### 3.2. “Pilot” tree and the representative isolates

**Figure 3:**
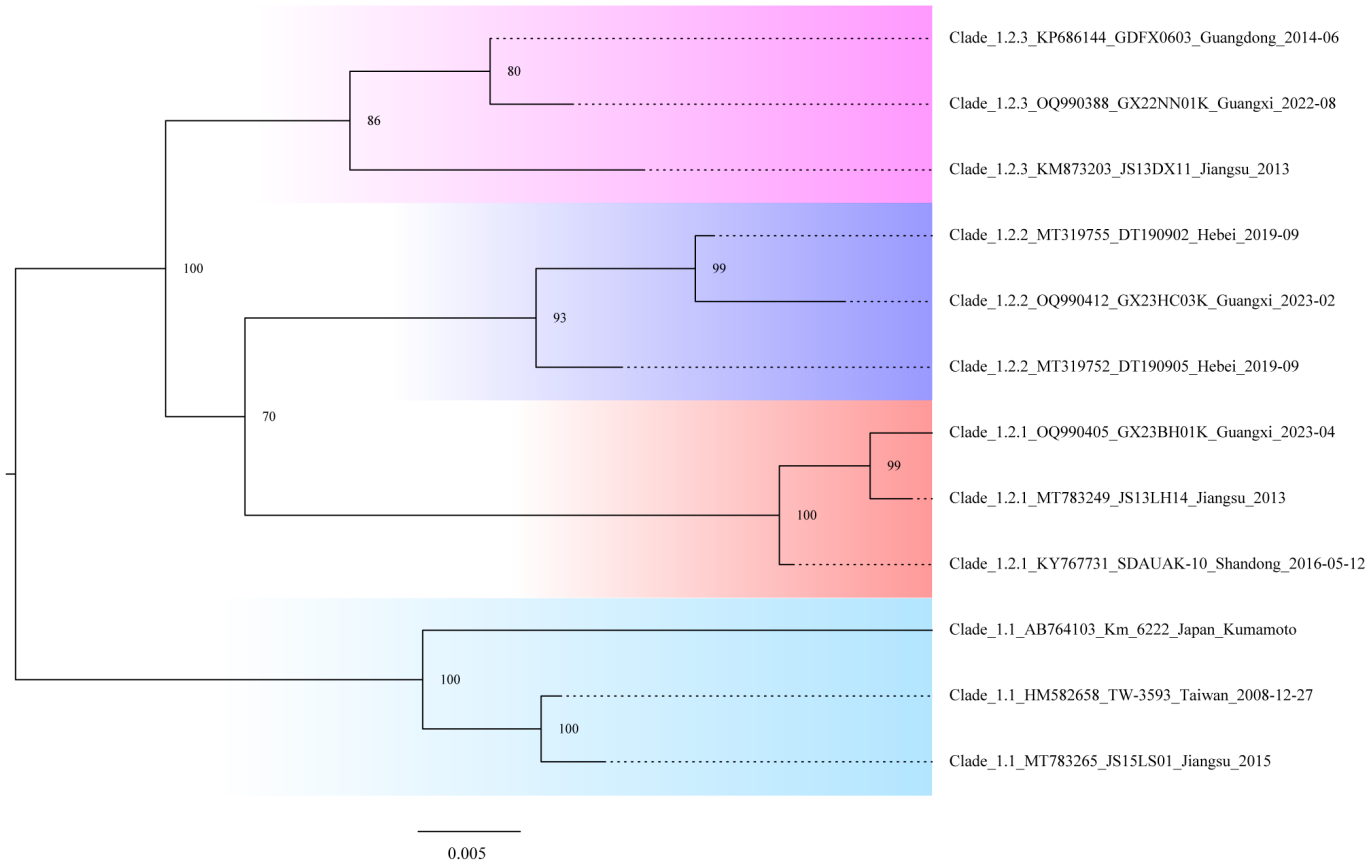
Classification “pilot” tree. Phylogenetic analysis is based on the gp85 genes of the selected isolates representing the second-order and third-order clades.

In the study, we needed to find a rapid, preliminary method for clade identification of the new isolates, using fewer sequences to construct evolutionary trees that are consistent with the topology of evolutionary trees constructed from the larger sequence datasets and significantly reduce the time to construct the evolutionary trees. To identify the second-order clades and the third-order clades of Clade 2, ML phylogenetic reconstruction was carried out using the 12 representative viral sequences. The new isolates can be quickly preliminary classified by using this pilot dataset (Figure 3; Supplementary Table S5).

### 3.3. Analysis of amino acid sequences

It was interestingly noted that the aa residues in one of the several branches are obviously distinct. Aa residues ^70^AD^71^ make up the majority of Clade 1.1, ^70^AS^71^ are the majority of Clades 1.2.1 and 1.2.3, and ^70^G-^71^ are the majority of Clade 1.2.2 (Supplementary Figure S2). Notably, Clade 1 2.2 was discovered to have a deletion of an aa-residue in the gp85 gene, which was noticeably different from Clades 1.1, 1.2.1, and 1.2.3.

## 4. Discussion

So far, most strains of ALV-K are considered to be no or low pathogenic, as chickens infected with ALV-K do not exhibit clinical or subclinical symptoms. At the same time, there is a lack of attention and long-term targeted detection of the isolates in the field. However, previous studies in 2015 found enhanced replication capacity or increased pathogenicity of ALV-K isolates obtained from the clinical samples (15, 25). In recent years, researchers have focused on the genetic evolution and pathogenicity changes of ALV-K. Currently, it is not only prevalent in the Chinese Yellow-chicken breeds but also distributed in other countries chicken breeds in Asia (11, 26, 27). The lack of attention on ALV-K has led to it being easily ignored in routine testing, especially with the emergence of co-infection with different subgroups of ALV and/or MDV (28), to the detriment of the ALV eradication programs. ALV-K has increasingly been isolated from Chinese Yellow-chicken recently, but there are few studies focused on the phylogenetic analysis of ALV-K isolates, and this study is a new attempt.

The study established a detailed classification approach for the purpose of more effectively tracking the source of ALV-K. Referring to the classification and nomenclature of Deng et al. (19, 20), we are able to trace the virus’s origin in the study because of the improvements in the accuracy of our ALV-K classification results. For example, sequence alignment was performed using MAFFT software, the gp85 gene sequence was compared with the reference strain TW-3593, and the identical repeated sequence was removed using BioEdit software, which will facilitate the construction of a topologically stable evolutionary tree.

The study found that the currently identified ALV-K strains could be divided into two second-order clades (Clades 1.1 and 1.2) and three third-order clades (Clades 1.2.1, 1.2.2, and 1.2.3) (Figure 2), based on phylogenetic analysis and genetic distance analysis. Clade 1.1 includes not only Chinese isolates from various sources but also Japanese strains. Clade 1.2 is a branch of strains that all originated in China. The phylogenetic trees of Clade 1.2 showed the ALV-K strains fell into 3 sub-subclades (Clades 1.2.1, 1.2.2, and 1.2.3). During the years 2022–2023, our group was successful in isolating 25 ALV-K isolates by detecting the P27 antigen of ALV on DF-1 cell cultures that had been inoculated with the tissue samples of the clinical diseased chickens and the plasma samples from birds in the routine eradication testing. The fact that the majority of the clinical bird isolates (11/14) and the routine eradication testing isolates (10/11) belonged to Clade 1.2 demonstrated that incomplete eradication of ALV is one of the primary reasons for getting these ALV-K. ALV-K is a vertically transmitted virus that is extremely difficult to eradicate once infected. For routine eradication and diagnostic assays, the presence of ALV-K is not easily detected due to its slow replication compared to other subgroups (12, 14) and the fact that it is only cultured on cells for one passage, thus making it easy to miss detection. These also suggest that we should pay attention to ALV-K monitoring when conducting “eradication” tests. In the analysis of aa residues on the gp85 sequence, the differences between Clades 1.1, 1.2.1, 1.2.2, and 1.2.3 were explained. Notably, Clade 1.2.2 was found to have a deletion of an aa-residue (^70^G-^71^) in the gp85 gene, and that was significantly different from Clades 1.1, 1.2.1, and 1.2.3. It is therefore assumed that Clade 1.1 virus was endemic in chickens in Taiwan, China, and Japan for years, leading to certain site changes and evolved into Clade 1.2, which was better adapted and led to endemicity in the flocks. In addition, we found a widespread co-infection of ALV-K and ALV-J in chickens during the ALV eradication process (Source unpublished data from our group).

In conclusion, we first proposed the definition of the current ALV-K strains based on the gp85 gene that were divided into two second-order clades (Clades 1.1 and 1.2) and three third-order clades (Clades 1.2.1, 1.2.2, and 1.2.3), and found that only two clades (Clades 1.1 and 1.2.3) are co-prevalent in Japan, while two new clades (1.2.1, 1.2.2) are co-prevalent in China. This system includes comprehensive criteria for the classification of new ALV-K isolates. We defined representative strains of each clade for the first time. Notably, Clade 1.2.2 was found to have a deletion of an amino acid residue in the gp85 gene, which was significantly different from Clades 1.1, 1.2.1, and 1.2.3. Our classification can play an irreplaceable role in tracking the evolution of ALV-K and provides a theoretical basis for enhanced epidemiological surveillance and eradication measures for ALV-K in the future.

## Data availability statement

The datasets presented in this study can be found in online repositories. The names of the repository/repositories and accession number(s) can be found in the article/Supplementary material.

## Ethics statement

The study focused on ALV-K using a modern molecular approach. The study was approved by the Animal Welfare and the Animal Experimental Ethical Committee of Guangxi University.

## Author contributions

JG and QD completed the data analysis and contributed to the experiment. WZ, FF, LL, and TW assisted in this experiment. PW provided the funding of research, reviewed, and approved the final manuscript. All authors contributed to the article and approved the submitted version.

## Funding

This work was supported by the Guangxi Program for Modern Agricultural Industry Technical System Construction-Chicken Industry [nycytxgxcxtd-19-03].

## Conflict of interest

The authors declare that the research was conducted in the absence of any commercial or financial relationships that could be construed as a potential conflict of interest.

## Publisher’s note

All claims expressed in this article are solely those of the authors and do not necessarily represent those of their affiliated organizations, or those of the publisher, the editors and the reviewers. Any product that may be evaluated in this article, or claim that may be made by its manufacturer, is not guaranteed or endorsed by the publisher.

## Footnotes

^1^ http://www.lonsera.cn/

^2^ https://blast.ncbi.nlm.nih.gov/Blast.cgi

^3^NCBI, http://www.ncbi.nlm.nih.gov/.
