# Supplementary material for: The phylogenetic analysis of the new emerging ALV-K revealing the co-prevailing of multiple clades in chickens and a proposal for the classification of ALV-K
Source: Front Vet Sci. 2023 Jul 28;10:1228109. doi: 10.3389/fvets.2023.1228109 (PMC10416628; doi:10.3389/fvets.2023.1228109)
Supplement: Supplementary file 1 [file Data_Sheet_1.docx]

***Supplementary Material***

**The phylogenetic analysis of the new emerging ALV-K revealing the co-prevailing of multiple clades in chickens and a proposal for the classification of ALV-K**

**Jinhan Guo^1^, Qiaomu Deng^1,2^, Weiyu Zhu^1^, Fumei Fu^1^, Linmin Liu^1^, Tianchao Wei^1^, Ping Wei^1,*^**

*** Correspondence:** Dr., Prof. Ping Wei: pingwei8@126.com

**Table S1. Primers for PCR amplifications to detect ALVs**

| **NO.** | **Primer** | **Sequence (5’-3’)** | **Product length (bp)** |
| --- | --- | --- | --- |
| 1 | ALV-A | GGATGAGGTGACTAAGAAAG | 692 |
|  |  | AGAGAAAGAGGGGTGTCTAAGGAG |  |
| 2 | ALV-B | GGATGAGGTGACTAAGAAAG | 847 |
|  |  | ATGGACCAATTCTGACTCATT |  |
| 3 | ALV-C | GGATGAGGTGACTAAGAAAG | 860 |
|  |  | GAGGCCAGTACCTCCCACG |  |
| 4 | ALV-D | GGATGAGGTGACTAAGAAAG | 797 |
|  |  | ATCCATACGCACCACAGTATTCG |  |
| 5 | ALV-J | GGATGAGGTGACTAAGAAAG | 545 |
|  |  | CGAACCAAAGGTAACACACG |  |
| 6 | ALV-K | GAGCATTGACACGCTTTCAGATTGG | 220 |
|  |  | GCCCCCGACAGGCACGC |  |

| **Table S2. Details of ALV isolates used in the study from the GenBank** | | | | | | |
| --- | --- | --- | --- | --- | --- | --- |
| **No.** | **Subgroups** | **Accession no.** | **Isolates** | **Time** | **Locations** | **Hosts** |
| 1 | A | M37980 | RSA | ＊ | France | ＊ |
| 2 | A | KU375453 | SDAU14A1 | 2014-08 | Shandong, China | Chicken |
| 3 | B | HM446005 | SDAU09C2 | 2009-04 | Shandong, China | Chicken |
| 4 | C | J02342 | RSV-Prague | 1977 | USA | ＊ |
| 5 | D | D10652 | RSV-S-R-D | 1998 | USA | ＊ |
| 6 | E | AY013303 | ev-1 | 2000 | USA | Chicken |
| 7 | E | EF467236 | SDO5O1 | 2005 | Shandong, China | Chicken |
| 8 | E | KC610515 | ALVE-B9 | ＊ | Canada | Chicken |
| 9 | J | Z46390 | HPRS-103 | 1995 | UK | Chicken |
| 10 | J | MF461280 | GX14YYA1 | 2014 | Guangxi, China | Yellow-chicken |
| 11 | J | KX058878 | GX14HG04 | 2014 | Guangxi, China | Yellow-chicken |
| 12 | K | HM582658 | TW-3593 | 2008-12 | Taiwan, China | Chicken |
| 13 | K | MK941182 | GD1701 | 2017-09 | Guangdong, China | Chicken |
| 14 | K | MT783265 | JS15LS01 | 2015 | Jiangsu, China | Yellow broiler chicken |
| 15 | K | AB764105 | Km_6343 | 2015 | Japan | Domesticus chicken |
| 16 | FGV | AB617819 | Sp-40 | 2011 | Japan | Chicken |
| 17 | K | KU605774 | GD14LZ | 2014 | Guangdong, China | Yellow-chicken |
| 18 | ＊ | AB682778 | Km_5892 | 2010 | Japan | Native chicken |
| 19 | ＊ | AB764106 | Km_6349 | ＊ | Japan | Domesticus chicken |
| 20 | ＊ | AB764101 | Km_6202 | ＊ | Japan | Domesticus chicken |
| 21 | ＊ | AB764101 | Km_6181 | ＊ | Japan | Domesticus chicken |
| 22 | FGV | AB617820 | Sp-53 | ＊ | Japan | Chicken |
| 23 | ＊ | ON840109 | KmN_77_clone_B | 2020 | Japan | Chicken |
| 24 | ＊ | _AB764104 | Km_6249 | ＊ | Japan | Domesticus chicken |
| 25 | ＊ | AB764103 | Km_6222 | ＊ | Japan | Domesticus chicken |
| 26 | ＊ | MN689736 | KmN_1 | 2017 | Japan | Chicken |
| 27 | ＊ | MW730795 | KmN_12 | 2018 | Japan | Chicken |
| 28 | K | MT783257 | JS15AJH02 | 2015 | Jiangsu, China | Yellow-chicken |
| 29 | K | MT783252 | JS14HX01 | 2014 | Jiangsu, China | Yellow-chicken |
| 30 | E/K | MT319753 | DT190904 | 2019-09 | Hebei, China | Yellow-chicken |
| 31 | K | MT783249 | JS13LH14 | 2013 | Jiangsu, China | Yellow-chicken |
| 32 | K | MT624730 | JS13LH14 | 2013 | Jiangsu, China | Yellow-chicken |
| 33 | ＊ | KF738251 | SD110503R | 2011-05 | Shandong, China | Chicken |
| 34 | ＊ | KY767731 | SDAUAK-10 | 2016-05 | Shandong, China | Chicken |
| 35 | K | MT624729 | JS15SG01 | 2015 | Jiangsu, China | Local chicken |
| 36 | K | MT783272 | JS15SG01 | 2015 | Jiangsu, China | Local chicken |
| 37 | K | KP686143 | GDFX0602 | 2014-06 | Guangdong, China | Yellow broiler chicken |
| 38 | ＊ | KM873219 | JS13LY15 | 2013 | Jiangsu, China | Yellow-chicken |
| 39 | K | KP686144 | GDFX0603 | 2014-06 | Guangdong, China | Yellow broiler chicken |
| 40 | K | MT783277 | JS16LH01 | 2016 | Jiangsu, China | Yellow-chicken |
| 41 | K | KP686142 | GDFX0601 | 2014-06 | Guangdong, China | Yellow broiler chicken |
| 42 | K | MT783273 | JS15YJ01 | 2015 | Jiangsu, China | Yellow-chicken |
| 43 | K | MT783268 | JS15LY02 | 2015 | Jiangsu, China | Yellow-chicken |
| 44 | K | MT783274 | JS15YJ02 | 2015 | Jiangsu, China | Yellow-chicken |
| 45 | ＊ | KM873209 | JS13LY03 | 2013 | Jiangsu, China | Chinese indigenous breeds |
| 46 | K | MT783283 | JS17DX02 | 2013/2017 | Jiangsu, China | Yellow-chicken |
| 47 | ＊ | KM873217 | JS13LY12 | 2013 | Jiangsu, China | Chinese indigenous breeds |
| 48 | ＊ | KM873218 | JS13LY13 | 2013 | Jiangsu, China | Chinese indigenous breeds |
| 49 | ＊ | KM873211 | JS13LY05 | 2013 | Jiangsu, China | Chinese indigenous breeds |
| 50 | ＊ | KM873220 | JS13LY16 | 2013 | Jiangsu, China | Chinese indigenous breeds |
| 51 | ＊ | KM873202 | JS13DX10 | 2013 | Jiangsu, China | Chinese indigenous breeds |
| 52 | ＊ | KM873203 | JS13DX11 | 2013 | Jiangsu, China | Chinese indigenous breeds |
| 53 | ＊ | AB670312 | Km_5844 | 2010 | Japan | Native chicken |
| 54 | ＊ | AB670314 | Km_5845 | 2010 | Japan | Native chicken |
| 55 | ＊ | AB669897 | Km_5943 | 2010 | Japan | Native chicken |
| 56 | E/K | KY490696 | JS14CZ02 | 2014 | Jiangsu, China | Chicken |
| 57 | K | MT783261 | JS15HNM01 | 2015 | Jiangsu, China | Yellow-chicken |
| 58 | ＊ | KF999961 | JS13-DX5 | 2013-06 | Jiangsu, China | Chicken |
| 59 | K | MT783282 | JS17DX01 | 2013/2017 | Jiangsu, China | Chicken |
| 60 | ＊ | KM873210 | JS13LY04 | 2013 | Jiangsu, China | Chinese indigenous breeds |
| 61 | K | OP035379 | SD20LH01 | 2020-06 | Shandong, China | Chicken |
| 62 | ＊ | KM873208 | JS13LH01 | 2013 | Jiangsu, China | Chinese indigenous breeds |
| 63 | ＊ | KF999962 | JS13-LH1 | 2013-06 | Jiangsu, China | Layer chicken |
| 64 | E/K | MT319752 | _DT190905 | 2019-09 | Hebei, China | Yellow-chicken |
| 65 | K | MT783262 | JS15HNM02 | 2015 | Jiangsu, China | Chicken |
| 66 | E/K | MT319755 | DT190902 | 2019-09 | Hebei, China | Yellow-chicken |
| 67 | E/K | MT319754 | DT190903 | 2019-09 | Hebei, China | Yellow-chicken |
| 68 | K | MT783250 | JS14BE01 | 2014 | Jiangsu, China | Chicken |
| 69 | E/K | MT319756 | DT190901 | 2019-09 | Hebei, China | Yellow-chicken |
| 70 | ＊ | KF746200 | JS11C1 | 2012 | Jiangsu, China | Domesticus chicken |
| 71 | K | KY581580 | HB2015032 | 2015-12 | Hubei, China | Chicken |
| 72 | E/K | KY490695 | JS14CZ01 | 2014 | Jiangsu, China | Chicken |
| 73 | K | MG770235 | JS13LY19 | 2014 | Jiangsu, China | Chicken |
| 74 | K | MT783258 | JS15BE01 | 2015 | Jiangsu, China | Chicken |
| 75 | K | MT783253 | JS14LH01 | 2014 | Jiangsu, China | Chicken |

**Table S3. Our ALV-K isolated and one to one corresponding the most similar reference strain in GenBank.**

| **Isolates** | ***env* gene length (bp)** | **Most similar known strain** | **Similarity (%)** | **Time** | **Location** |
| --- | --- | --- | --- | --- | --- |
| GX22NN01K | 1617 | GDFX0602_KP686143 | 99.57 | 2014 | Guangdong |
| GX22NN02K | 1617 | GD1701_MK941182 | 99.51 | 2017 | Guangdong |
| GX22YL10K | 1617 | GD1701_MK941182 | 99.38 | 2017 | Guangdong |
| GX22GL02K | 1614 | GDFX0602_KP686143 | 98.95 | 2014 | Guangdong |
| GX22GL01K | 1617 | GDFX0603_KP686144 | 98.89 | 2014 | Guangdong |
| GX23BH01K | 1617 | DT190904_MT319753 | 99.75 | 2019 | Hebei |
| GX23BH02K | 1617 | DT190904_MT319753 | 99.69 | 2019 | Hebei |
| GX23BH03K | 1617 | DT190904_MT319753 | 99.81 | 2019 | Hebei |
| GX23BH04K | 1617 | DT190904_MT319753 | 99.26 | 2019 | Hebei |
| GX23BH05K | 1614 | HB2015032_KY581580 | 99.38 | 2015 | Hubei |
| GX22YL01K | 1614 | JS14BE01_MT783250 | 98.89 | 2014 | Jiangsu |
| GX22YL02K | 1614 | JS14BE01_MT783250 | 99.63 | 2014 | Jiangsu |
| GX23HC01K | 1617 | GDFX0602_KP686143 | 99.26 | 2014 | Guangdong |
| GX23HC02K | 1617 | DT190904_MT319753 | 99.63 | 2019 | Hebei |
| GX23HC03K | 1614 | HB2015032_KY581580 | 99.38 | 2015 | Hubei |
| GX22GG01K | 1614 | DT190903_MT319754 | 99.57 | 2019 | Hebei |
| GX22GG03K | 1617 | JS14BE01_MT783250 | 98.02 | 2014 | Jiangsu |
| GX22GG04K | 1614 | GD1701_MK941182 | 98.39 | 2017 | Guangdong |
| GX23GG01K | 1617 | GDFX0602_KP686143 | 99.07 | 2014 | Guangdong |
| GX23GG02K | 1614 | HB2015032_KY581580 | 99.32 | 2015 | Hubei |
| GX23GG03K | 1614 | JS14BE01_MT783250 | 99.13 | 2014 | Jiangsu |
| GX22BL01K | 1617 | GD1701_MK941182 | 99.20 | 2017 | Guangdong |
| GX23NN01K | 1617 | JS16LH01_MT783277 | 99.69 | 2016 | Jiangsu |
| GX22GG10K | 1614 | JS14BE01_MT783250 | 99.38 | 2014 | Jiangsu |
| GX22YL11K | 1614 | JS14BE01_MT783250 | 99.63 | 2014 | Jiangsu |

**Table S4. Estimates of the evolutionary distances between clades of ALV-K.**

| **Clades** | **No. of base substitutions per site** | | | |
| --- | --- | --- | --- | --- |
|  | Clade 1.1 | Clade1.2.1 | Clade 1.2.2 | Clade1.2.3 |
| Clade 1.1 |  |  |  |  |
| Clade 1.2.1 | 0.0656 |  |  |  |
| Clade 1.2.2 | 0.0599 | 0.0469 |  |  |
| Clade 1.2.3 | 0.0507 | 0.0459 | 0.0438 |  |

^a^ The number of base substitutions per site from averaging over all sequence pairs between clades are shown. Analyses were conducted using the Maximum Composite Likelihood model (1). The rate variation among sites was modeled with a gamma distribution (shape parameter = 1). This analysis involved 87 nucleotide sequences. Codon positions included were 1st+2nd+3rd+Noncoding. All ambiguous positions were removed for each sequence pair (pairwise deletion option). There were a total of 1008 positions in the final dataset. Evolutionary analyses were conducted in MEGA 11 (2). Different first-order clades have an average distance per site above 10% (0.1). Different second-order clades have an average distance per site above 5% (0.05). Different third-order clades have an average distance per site above 2.5% (0.025).

1. Tamura K, Nei M, Kumar S. Prospects for Inferring Very Large Phylogenies by Using the Neighbor-Joining Method. Proceedings of the National Academy of Sciences of the United States of America (2004) 101(30):11030-5. Epub 2004/07/20. doi: 10.1073/pnas.0404206101.

2. Tamura K, Stecher G, Kumar S. Mega11: Molecular Evolutionary Genetics Analysis Version 11. Molecular biology and evolution (2021) 38(7):3022-7. Epub 2021/04/24. doi: 10.1093/molbev/msab120.

**Table S5. Details of the representative viruses in the pilot tree of the clades.**

| **Clades** | **Representative viruses (Accession no.)** |
| --- | --- |
| 1.1 | TW-3593(HM582658), Km_6222(AB764103), JS15LS01(MT783265) |
| 1.2.1 | JS13LH14(MT783249), GX23BH01K(OQ990405), SDAUAK-10(KY767731) |
| 1.2.2 | DT190902(MT319755), DT190905(MT319752), GX23HC03K(OQ990412) |
| 1.2.3 | GX22NN01K(OQ990388), GDFX0603(KP686144), JS13DX11(KM873203) |


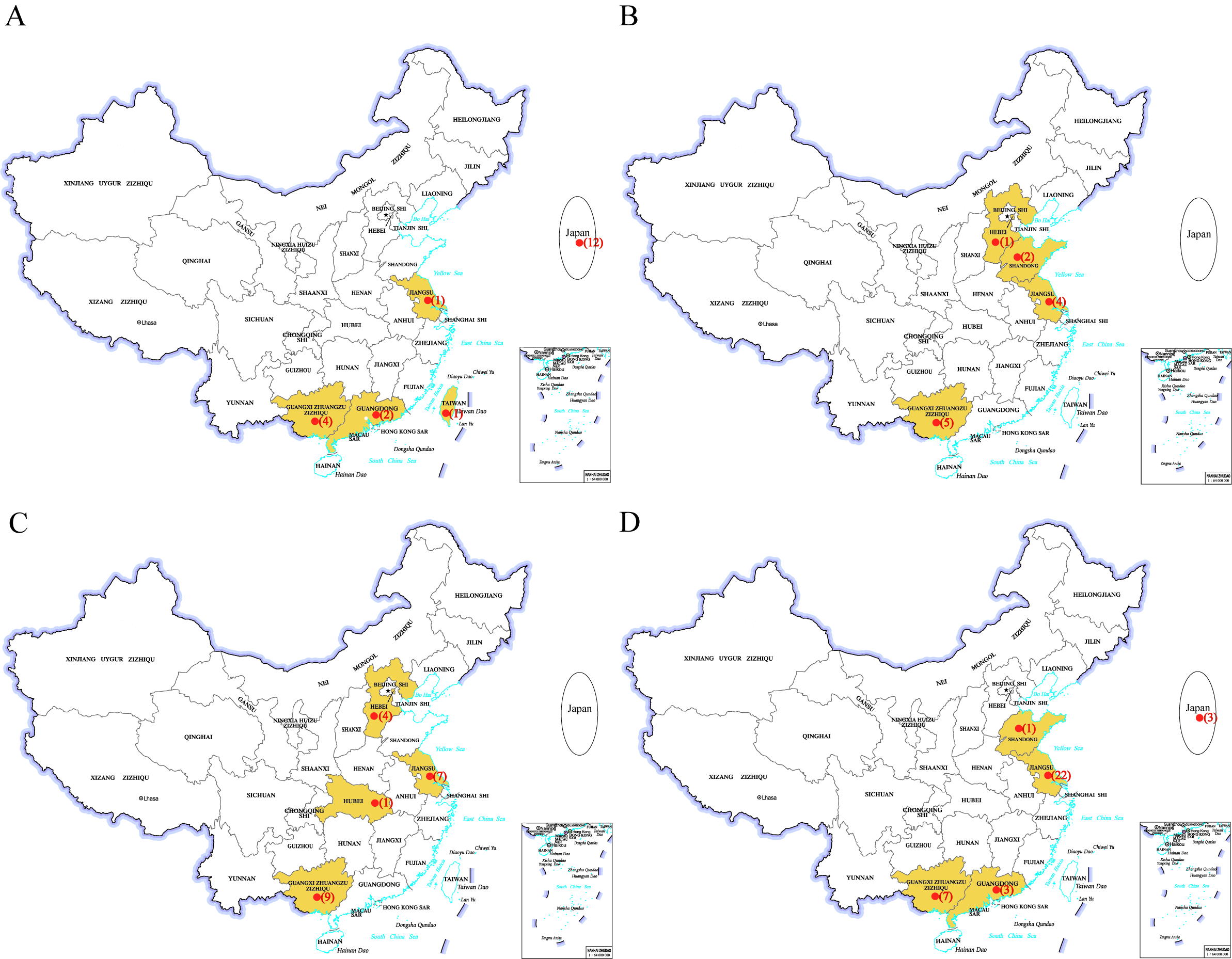


**Figure S1. Geographical distribution of isolates in different clades.** A, Clade 1.1; B, Clade 1.2.1; C, Clade 1.2.2; D, Clade 1.2.3.


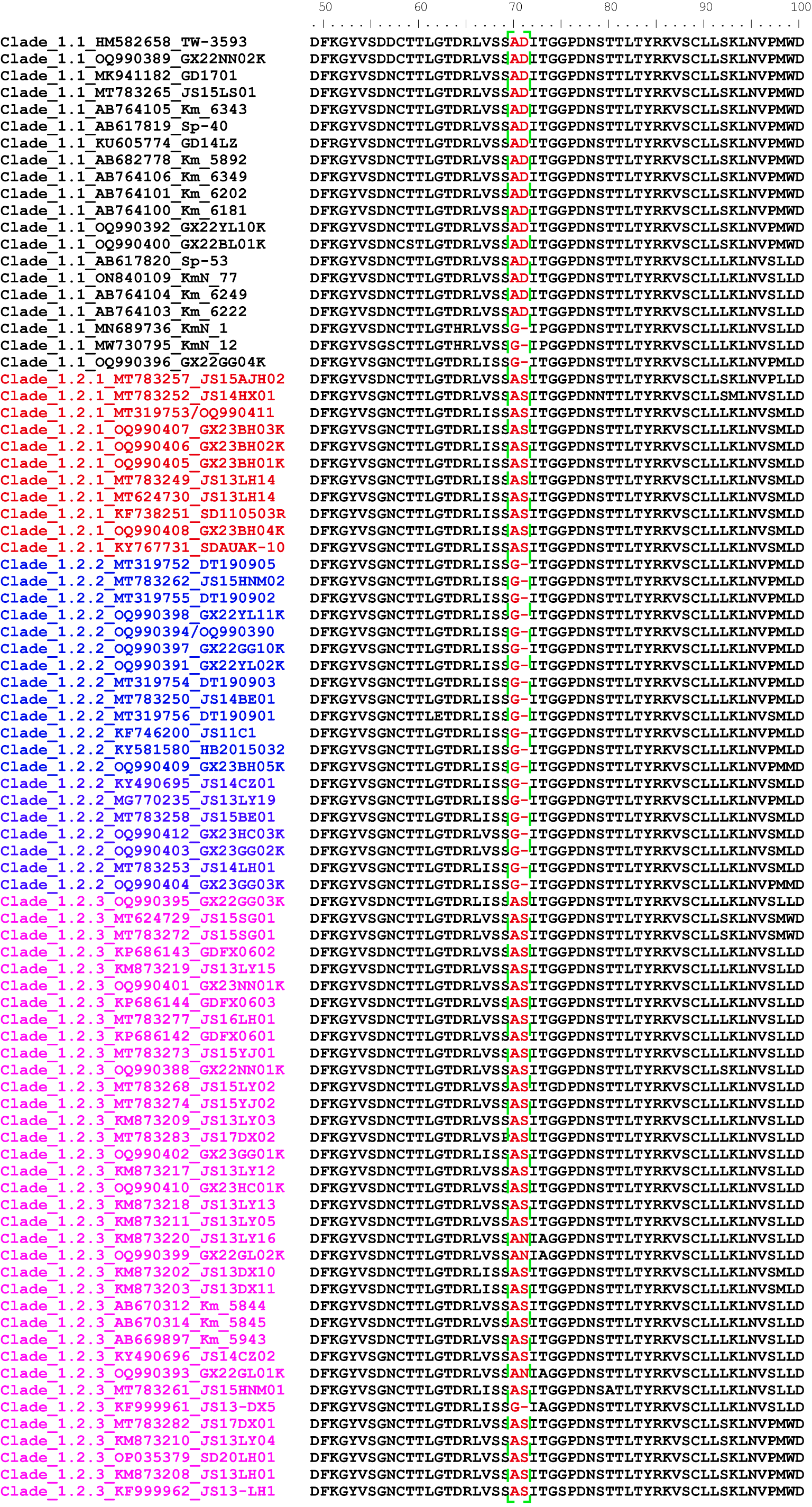


**Figure S2. Alignment of the aa sequences of the gp85 protein of our 25 isolates with other representative strains.** Important residues are marked by dashed boxes. The different colors (black, red, blue, purple) represent the Clade 1.1, 1.2.1, 1.2.2, and 1.2.3, respectively.
